# Increased Risk of Cardiovascular Death in Breast Cancer Patients Without Chemotherapy or (and) Radiotherapy: A Large Population-Based Study

**DOI:** 10.3389/fonc.2020.619622

**Published:** 2021-01-28

**Authors:** Tianwang Guan, Hanbin Zhang, Jinming Yang, Wenrui Lin, Kenie Wang, Miao Su, Weien Peng, Yemin Li, Yanxian Lai, Cheng Liu

**Affiliations:** ^1^Department of Cardiology, Guangzhou First People’s Hospital, South China University of Technology, Guangzhou, China; ^2^Department of Cardiology, Guangzhou First People’s Hospital, Guangzhou Medical University, Guangzhou, China; ^3^Department of Cardiology, Laboratory of Heart Center, Zhujiang Hospital, Southern Medical University, Guangzhou, China; ^4^Department of Clinical Medicine, Clinical Medical School, Guangzhou Medical University, Guangzhou, China; ^5^The First Department of Breast Cancer, Tianjin Medical University Cancer Institute and Hospital, National Clinical Research Center for Cancer, Tianjin, China

**Keywords:** breast cancer, cardiovascular death, chemotherapy, radiotherapy, cardio-oncology

## Abstract

**Background:**

Cardiovascular death (CVD) in breast cancer patients without chemotherapy (CT) or (and) radiotherapy (RT) has not been studied yet. This study evaluates the correlation between breast cancer and CVD risk independent of chemotherapy or (and) radiotherapy.

**Methods:**

Data of female breast cancer patients without receiving CT or RT were retrieved from the Surveillance, Epidemiology, and End Result (SEER) database (2004–2015). Data were divided into two cohorts: tumor resection cohort and no resection cohort. The CVD risk in patients was expressed as standardized mortality ratios (SMRs). A 1:1 propensity score matching (PSM) was applied to balance inter-group bias, and competing risk regressions were utilized to evaluate the impact of tumor resection on CVD.

**Results:**

The CVD risk was significantly higher (SMR = 2.196, 95% CI: 2.148–2.245, *P*<0.001) in breast cancer patients who did not receive CT or RT compared to the general population. Breast cancer patients without tumor resection showed higher CVD risk than patients who underwent tumour resection (tumor resection SMR = 2.031, 95% CI: 1.983–2.079, *P*<0.001; no resection SMR = 5.425, 95% CI: 5.087–5.781, *P*<0.001). After PSM, the CVD risk among patients without tumor resection indicated an increase of 1.165-fold compared to patients with tumor resection (HR=1.165, 95% CI: 1.039–1.306, *P*=0.009).

**Conclusions:**

Female breast cancer patients are at higher risk of CVD despite unexposure to cardio-toxic CT or RT. However, female breast cancer patients subjected to tumor resection have decreased CVD risk. These results indicated that monitoring female breast cancer patients not receiving RT or CT might serve as a preventative measure against CVD.

## Introduction

Breast cancer is a serious common threat for women’s health, accounting for 30% of new cancer cases in females, and is on a growing trend (1, 2). Rapid advances in cancer screening and treatment technologies increased the five-year survival rate to 90% (1). By 2014, the estimated number of breast cancer survivors has reached over 3.1 million in the United States and is anticipated to rise to more than 3.9 million by 2024 (3). However, cardiovascular diseases appear to be a life-threatening complication for the survivors. Cardiovascular death (CVD) is becoming the leading cause of death in breast cancer survivors (4, 5). Identifying the population at high risk of CVD is a key step in implementing routine preventative measures to improve breast cancer survivors’ prognosis.

At the present, the mainstream belief among cardio-oncologists is that cardiotoxic therapies, including chemotherapy (CT) or radiotherapy (RT), are the main contributors to increase CVD risk in breast cancer survivors (6, 7). The clinical guideline of American Society of Clinical Oncology (ASCO) (8) limits the target population needing prevention and monitoring of cardiac dysfunction to those who have accepted cardiotoxic therapies. However, the target population mentioned in ASCO is restricted, and the guideline might neglect CVD risk among cancer patients who opted out of cardiotoxic therapies.

Previous studies indicated that CVD risk might also be higher among breast cancer patients not exposed to CT or RT. A recent study revealed that breast cancer survivors suffered elevated CVD risk (9). Furthermore, a population-based study also showed that CVD risk of breast cancer survivors was apparently higher than that of the general population (10). These two studies evaluated CVD risk in breast cancer survivors regardless of the treatment regimen (with or without CT/RT). Surprisingly, another study reported no increase in CVD risk among breast cancer survivors pretreated with CT/RT compared to the general population (11). These results suggested that elevated CVD risk among breast cancer survivors was resulting in part from those not exposed to CT or RT. A single-center study supported this possibility and found decreased cardiac function among cancer patients not exposed to CT or RT (12). Another study also revealed that tumors could directly induce cardiovascular damage by tumor-induced inflammation in tumor-bearing mice (13). The cardiotoxicity of CT and RT in breast cancer patients is confirmed, but it remains elusive whether avoiding CT and/or RT regimens lower or increase CVD risk in breast cancer survivors. This calls for urgent need to evaluate, by large-scale population-based study, the CVD risk among breast cancer patients who opted out of the CT and/or RT treatments.

On the molecular level, breast cancer is a heterogeneous disease and the treatment selections for breast cancer patients are affected by the molecular subtypes, especially CT and hormonal therapy (14). Breast cancer patients who received neither CT nor RT may receive hormonal treatments. Hormonal treatments in breast cancer mainly include selective estrogen receptor modulators (SERMs), aromatase inhibitors (AIs) and ovarian suppression, and their cardiovascular effects are heterogeneity. Firstly, tamoxifen and other SERMs increase the risk of venous thromboembolism, but improve the outcomes of atherothrombosis related cardiovascular events in patients with breast cancer (15–18). Secondly, AIs are associated with increased risks of CVD and low-density lipoprotein cholesterol serum levels compared to tamoxifen (19, 20). However, more studies on AIs showed that AIs are related to higher risks of cardiovascular events compared to tamoxifen, possibly due to tamoxifen related cardioprotective effects, and AIs themselves may have no effects on cardiovascular events and cholesterol levels compared to placebo or no treatment (16–18). Thirdly, there is no evidence that ovarian suppression has cardiovascular specific side effects in breast cancer patients (21). These results suggested that hormonal treatments might add minor effect (possibly no effects or cardioprotective effects) on CVD risk in breast cancer patients who received neither CT nor RT.

This study evaluates the CVD risk among breast cancer patients who opted out of the CT or (and) RT, no matter they received hormonal treatments or not. The analysis aims to achieve three objectives. Objective 1 is to evaluate CVD risk of breast cancer patients who never received CT or RT in comparison to the general population. Objective 2 is to evaluate CVD risk of breast cancer patients who never received CT or RT and were either subjected or not to tumor resection in comparison to the general population. Objective 3 is to conduct internal comparisons among breast cancer patients, aiming to reduce treatment selection bias and evaluate the independent effect of tumor removal on the CVD risk in breast cancer patients who never received CT or RT. The study aims at updating the guidelines of which breast cancer population requires prevention and monitoring for CVD risk.

## Patients and Methods

### Data Source

In this registry-based cohort study, the data of breast cancer patients were obtained from the Surveillance, Epidemiology, and End Result database (SEER) database, an authoritative program providing data of 18 cancer registries and covering approximately 34.6% of the American population (http://seer.cancer.gov/). The SEER database has been frequently used for cardio-oncology studies (9, 10, 22, 23). To compare with the cohort derived from the SEER database, the referred standardized population cohort was retrieved from Wide-ranging Online Data for Epidemiologic Research of the Centers for Disease Control and Prevention (CDC WONDER). CDC WONDER shares data of the general American population, which is based on death certificates of American residents. Ethical approval of this publicly available information was not required (9).

### Study Population and Design

**Figure 1:**
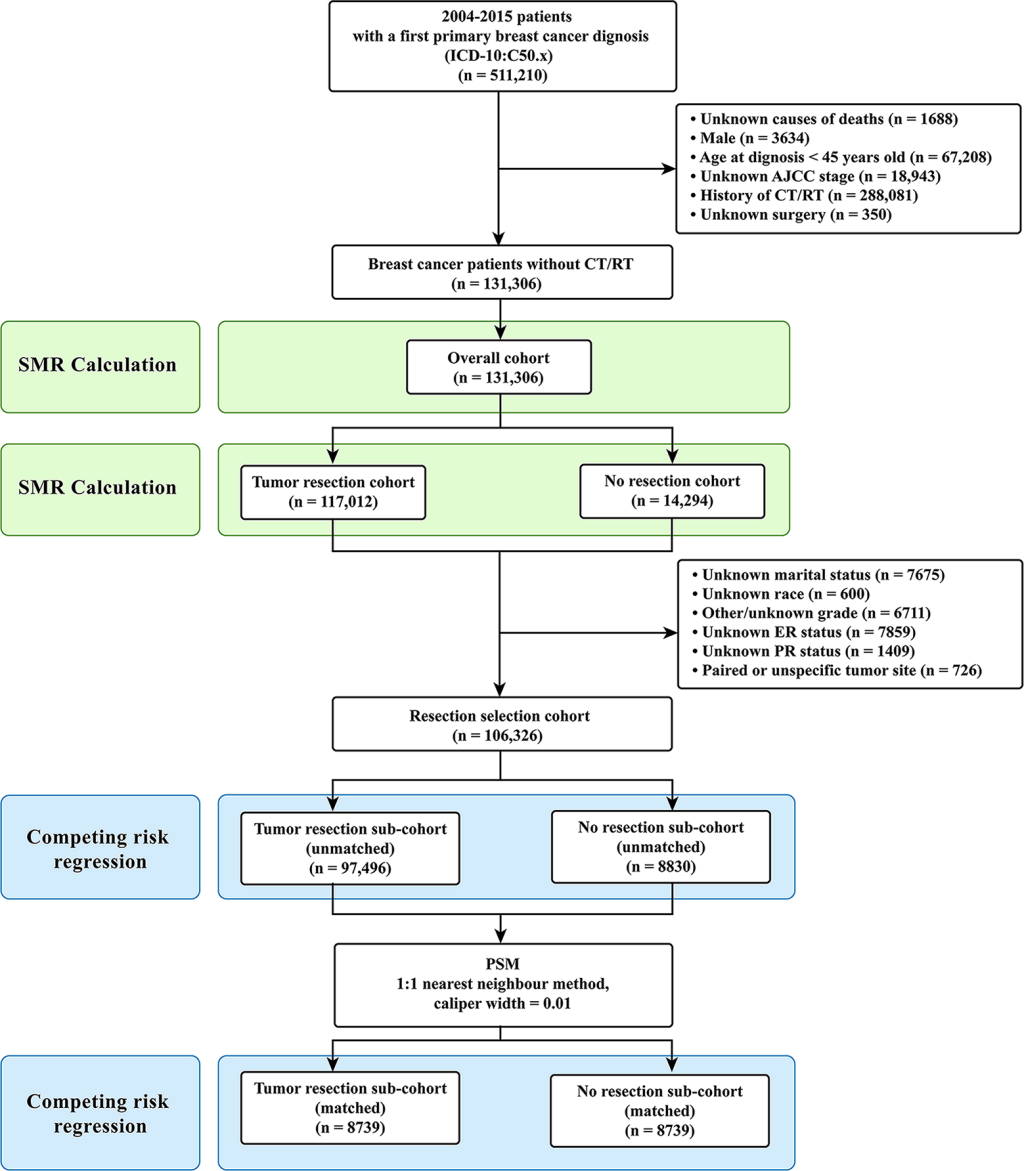
Selection of eligible patients and study design. CVD, cardiovascular death; SMR, standardized mortality ratio.

Only the female breast cancer patients without evidence for chemotherapy (CT) or radiotherapy (RT) were included in this study. Targeted therapy is classified as one kind of CT in the SEER database. The inclusion criteria were as follows: (1) case selection (site and morphology, primary site-labeled) = “C50.x”; (2) pathological diagnosis between 2004 and 2015; (3) patients with only one primary tumor; (4) patients with active follow-up. The exclusion criteria were as follows: (1) history of CT or RT; (2) unknown causes of deaths; (3) male patients; (4) age at diagnosis under 45 years old; (5) unknown stage according to the American Joint Committee on Cancer (AJCC) staging system; (6) unknown surgery. Patients under 45 years old were excluded due to their very low numbers (**Figure 1**) (22). After the inclusion and the exclusion criteria, we established an overall cohort.

Firstly, for Objective 1, CVD risk in 10-year age-stratum (45-54, 55-64, 65-74, 75-84, ≥85) of the overall cohort was compared to that of the age-matched U.S. female population, which was expressed as standardized mortality ratio (SMR). Secondly, for Objective 2, the overall cohort was categorized into two cohorts according to surgical resection: tumor resection cohort and no resection cohort. We compared the CVD risk between the tumor resection cohort and the no resection cohort by using SMR. Thirdly, for Objective 3, participants were further excluded from the overall patients if they had unknown or unspecific information regarding their marital status, race, grade, estrogen receptor (ER) status, and progesterone receptor (PR) status. Patients with paired tumor site or unspecific tumor site were also excluded owing to their very low number. Then resection selection cohort was extracted and further divided into two sub-cohorts: tumor resection sub-cohort and no resection sub-cohort (**Figure 1**).

### Participant Variables and Outcomes

Patient variables included surgery (tumor resection and no resection), age at diagnosis (45-60 years, >60 years), race (white, non-white), marital status (married, unmarried), laterality (left, right), grade (high, low), ER status (positive, negative), PR status (positive, negative), human epidermal receptor 2 (HER2) status (positive, negative, unknown), and AJCC stage (I, II, III, and IV) (4).

We classified causes of death as CVD or non-CVD. The CVD was defined as deaths due to heart disease (I00-I09, I11, I13, I20-I51), hypertension without heart disease (I10, I12), cerebrovascular disease (I60-I69), atherosclerosis (I70), aortic aneurysm and dissection (I71) and other diseases of arteries, arterioles and capillaries (I72-I78), according to the International Classification of Disease-10 (ICD-10) codes (22, 23). The non-CVD contained patients died from other causes and were considered as the competing events against CVD. Patients who survived till the last follow-up were treated as censored observations. The follow-up period was calculated as the time from the date of first diagnosis with breast cancer to the date of death or last follow-up. The final follow-up date was on December 31, 2015.

### Statistical Analysis

Chi-square test was used to evaluate categorical variables in baseline characteristics. SMR was defined as the ratio of the observed deaths to the expected (24). The expected number of deaths was calculated according to the following formula: expected deaths = person-years × mortality rate of CVD among general population, where the mortality rate of CVDs is available on CDC WONDER (23), and the person-years is the sum of patients’ survival time, from the date of breast cancer diagnosis to the date of study completion or the date of CVD. Ninety-five percent confidence intervals (95% CIs) and *P* value of SMRs were calculated by using the methods by Rothman-Boice (24) and by Altman et al. (25), respectively.

To reduce potential imbalance between breast patients who received tumor resection and no resection, a 1:1 propensity score matching (PSM) was applied. The PSM was performed using logistic regression and nearest neighbor method with caliper width of 0.01. PSM should match the confounding variables instead of all baseline variables (26). The potential confounding variables used for matching included age at diagnosis, marital status, grade, ER status, PR status, HER2 status, and AJCC stage. The balances between matched covariates were acceptable if *P* values were greater than 0.05 (27).

Further, the univariate and multivariate Fine and Gray’s competing risks regressions were used to evaluate the independent effect of tumor resection on the CVD risk among breast cancer patients without CT or RT. The Fine and Gray’s competing risks regression was performed to account for the two competing events: CVD deaths and non-CVD deaths.

Chi-square test and PSM were analyzed using SPSS version 25.0 (SPSS, Chicago, IL) and R software version 3.6.1 (https://www.r-project.org), respectively. Fine and Gray’s competing risks regression was conducted using Stata version 15 (Stata Corp, College Station, TX, USA). A two-tailed *P* value < 0.05 was considered statistically significant.

## Results

### Patient Selections and Baseline Characteristics

A total of 131,306 female breast cancer patients without CT or RT between 2004 and 2015 were included in this study, of whom the tumor resection cohort included 117,012 (89.1%) patients and the no resection cohort included 14,294 (10.9%) patients. After further selection, the resection cohort consisted of 106,326 breast cancer patients.

Compared to patients without tumor resection, patients with resection were more likely to be younger, married, white, ER-positive, PR-positive, HER2-unknown, and have lower grade, left breast cancer and have lower AJCC stage (**Supplementary Table S1**). The average follow-up time was 63.8 (range: 63.6-64.1) months among 131,306 breast cancer patients.

### The CVD Risk in Breast Cancer Patients and General Population

**Figure 2:**
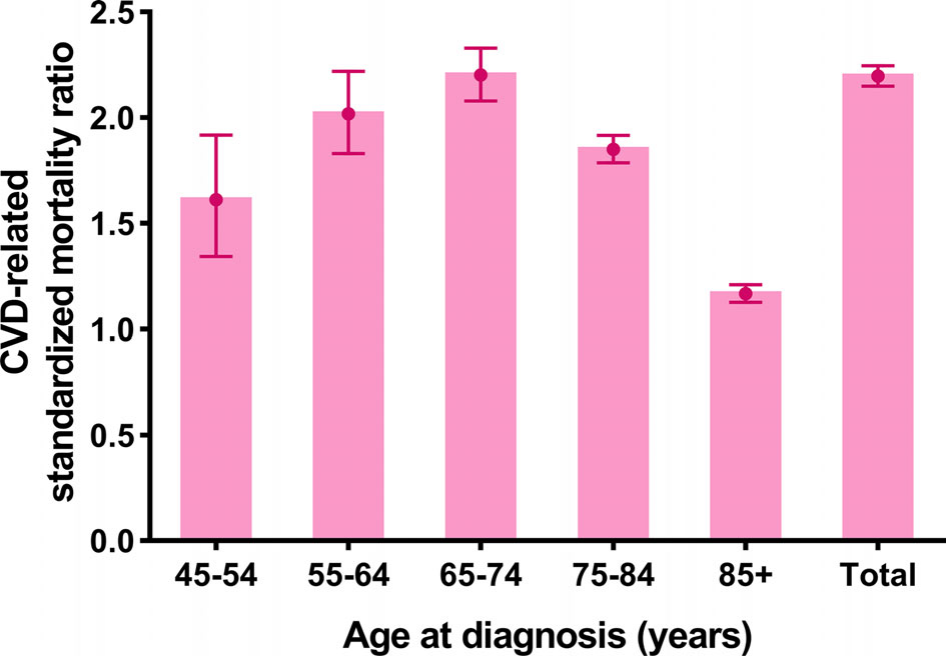
Cardiovascular death (CVD)-related standardized mortality ratios (SMRs) for breast cancer patients without chemotherapy or radiotherapy.

The CVD-related SMR was significantly higher in breast cancer patients without CT or RT (SMR = 2.196, 95% CI: 2.148–2.245, *P*<0.001) compared to the general population. In all age strata (≥45 years old), breast cancer patients without CT or RT had a higher CVD risk than the general population at the same age (all *P*<0.001) (**Figure 2** and **Supplementary Table S2**).

**Figure 3:**
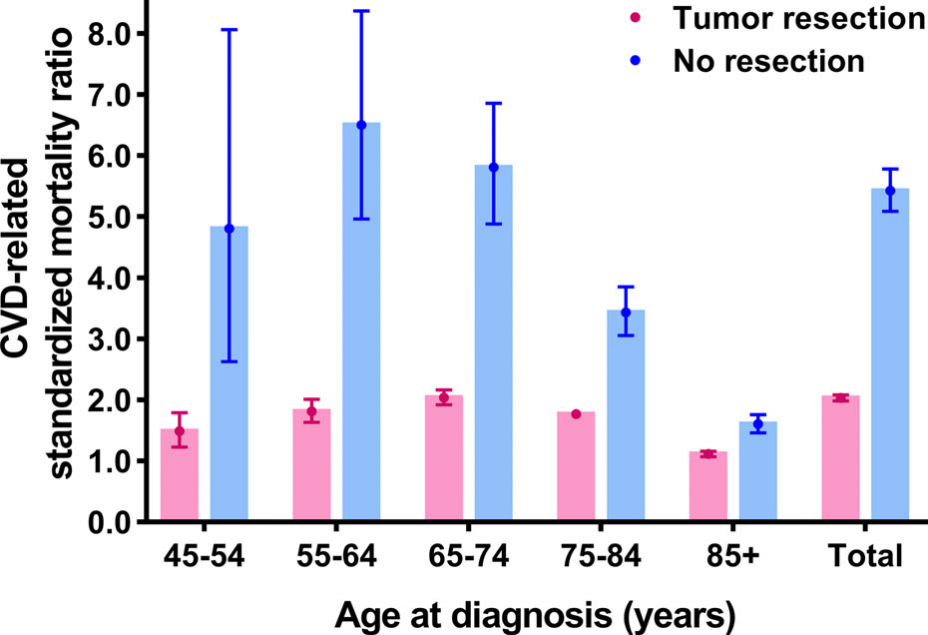
Cardiovascular death (CVD)-related standardized mortality ratios (SMRs) for breast cancer patients with and without tumor resection.

Further, CVD-related SMRs were increased to 2.031 folds (SMR = 2.031, 95% CI: 1.983–2.079, *P*<0.001) and 5.425 folds (SMR = 5.425, 95% CI: 5.087–5.781, *P*<0.001) in breast cancer patients with tumor resection and without resection respectively, compared to the general population. In all age strata (≥45 years old), the increased extent of CVD-related SMRs **were** all higher in patients without resection compared to the patients with tumor resection (no resection SMRs: 1.603–6.500; tumor resection SMRs: 1.113–2.037) (**Figure 3** and **Supplementary Table S2**).

### Propensity Score-Matched Analysis

Of the 106,326 breast cancer patients selected for internal comparisons, 97,496 (91.7%) received tumor resection and 8830 (8.3%) did not. Before PSM, the variables (age at diagnosis, race, marital status, grade, ER status, PR status, HER2 status, AJCC stage) showed imbalanced between the two groups (all *P*<0.05). After the 1:1 PSM, 17,478 patients were included in the matched cohort, and the confounding covariates became balanced between the two groups (**Table 1**). Laterality and race were not allocated in PSM, and the former was balanced but the later was imbalanced before and after PSM. However, race had no confounding effect on the results after PSM (**Table 2** and **Supplementary Table S4**).

**Table 1:** Baseline characteristics before and after propensity score matching.

Variable	Before PSM (N/%)			After PSM (N/%)		
	No resection	Tumor resection	*P*	No resection	Tumor resection	*P*
**N**	8830	97,496		8739	8739	
**Age at diagnosis**			< 0.001			0.688
45-60 years	2205 (25.0)	32,652 (33.5)		2197 (25.1)	2174 (24.9)	
> 60 years	6625 (75.0)	64,844 (66.5)		6542 (74.9)	6565 (75.1)	
**Race**			< 0.001			< 0.001
White	6737 (76.3)	80,127 (82.2)		6668 (76.3)	7079 (81.0)	
Non-White^a^	2093 (23.7)	17,369 (17.8)		2071 (23.7)	1660 (19.0)	
**Marital status**			< 0.001			0.610
Married	3014 (34.1)	49,752 (51.0)		2991 (34.2)	3023 (34.6)	
Unmarried	5816 (65.9)	47,744 (49.0)		5748 (65.8)	5716 (65.4)	
**Laterality**			0.173			0.193
Left	4587 (51.9)	49,909 (51.2)		4535 (51.9)	4449 (50.9)	
Right	4243 (48.1)	47,587 (48.8)		4204 (48.1)	4290 (49.1)	
**Grade**^b^			<0.001			0.961
Low	5940 (67.3)	73,488 (75.4)		5875 (67.2)	5878 (67.3)	
High	2890 (32.7)	24,008 (24.6)		2864 (32.8)	2861 (32.7)	
**ER status**			< 0.001			1.000
Positive	7504 (85.0)	85,372 (87.6)		7431 (85.0)	7431 (85.0)	
Negative	1326 (15.0)	12,124 (12.4)		1308 (15.0)	1308 (15.0)	
**PR status**			< 0.001			0.092
Positive	6410 (72.6)	74,900 (76.8)		6350 (72.7)	6250 (71.5)	
Negative	2420 (27.4)	22,596 (23.2)		2389 (27.3)	2489 (28.5)	
**HER2 status**			<0.001			0.296
Positive	868 (9.8)	4,525 (4.6)		798 (9.1)	788 (9.0)	
Negative	4979 (56.4)	46,354 (47.5)		4958 (56.7)	4870 (55.7)	
Unknown	2983 (33.8)	46,617 (47.8)		2983 (34.1)	3081 (35.3)	
**AJCC stage**			< 0.001			0.563
I	1958 (22.2)	58,956 (60.5)		1958 (22.4)	1908 (21.8)	
II	2455 (27.8)	31,204 (32.0)		2455 (28.1)	2505 (28.7)	
III and IV	4417 (50.0)	7336 (7.5)		4326 (49.5)	4326 (49.5)	

^a^Non-White includes Black/American Indian/Alaska Native and Asian/Pacific Islander.

^b^Low (Grade I: well differentiated and Grade II: moderately differentiated) and high (Grade III: poorly differentiated and Grade IV: undifferentiated).

ER, estrogen receptor; HER2, human epidermal receptor 2; PR, progesterone receptor; PSM, propensity score matching.

**Table 2:** Univariate competing-risks regression analysis of cardiovascular death.

Variable	Before PSM		After PSM	
	HR (95% CI)	*P* Value	HR (95% CI)	*P* Value
**Tumor resection**				
Yes	Reference		Reference	
No dissection	1.468 (1.345-1.603)	< 0.001	1.140 (1.017-1.279)	0.025
**Age at diagnosis**				
45-60 years	Reference		Reference	
> 60 years	12.789 (11.236-14.556)	< 0.001	8.444 (6.270-11.374)	< 0.001
**Race**				
White	Reference		Reference	
Non-White^a^	0.837 (0.781-0.898)	< 0.001	0.916 (0.791-1.062)	0.245
**Marital status**				
Married	Reference		Reference	
Unmarried	2.616 (2.475-2.765)	< 0.001	1.934 (1.681-2.226)	< 0.001
**Laterality**				
Left	Reference		Reference	
Right	0.964 (0.917-1.013)	0.147	0.930 (0.828-1.044)	0.220
**Grade**^b^				
Low	Reference		Reference	
High	0.955 (0.903-1.010)	0.109	0.882 (0.778-1.000)	0.051
**ER status**				
Positive	Reference		Reference	
Negative	0.984 (0.915-1.058)	0.664	0.849 (0.714-1.010)	0.065
**PR status**				
Positive	Reference		Reference	
Negative	1.054 (0.996-1.115)	0.070	0.831 (0.727-0.950)	0.007
**HER2 status**				
Positive	Reference		Reference	
Negative	0.915 (0.778-1.077)	0.286	0.989 (0.761-1.286)	0.935
Unknown	1.265 (1.081-1.480)	0.003	1.560 (1.210-2.013)	0.001
**AJCC stage**				
I	Reference		Reference	
II	1.445 (1.370-1.525)	< 0.001	1.139 (0.972-1.335)	0.109
III and IV	1.444 (1.334-1.562)	< 0.001	0.869 (0.748-1.009)	0.065

^a^Non-White includes Black/American Indian/Alaska Native and Asian/Pacific Islander.

^b^Low (Grade I: well differentiated and Grade II: moderately differentiated) and high (Grade III: poorly differentiated and Grade IV: undifferentiated).

ER, estrogen receptor; HER2, human epidermal receptor 2; HR, hazard ratios; PR, progesterone receptor; PSM, propensity score matching.

### Competing Risk Regressions in Internal Comparisons

In univariate analysis before PSM and after PSM, tumor resection, age at diagnosis, marital status, PR status, and HER2 status were correlated with CVD among breast cancer patients (**Table 2**). The breast cancer patients without tumor resection showed increased CVD risk compared to the patients with tumor resection (after PSM, unadjusted HR = 1.140, 95% CI: 1.017–1.279, *P*=0.025). To eliminate the possibility of producing false positive results, regression analysis of multivariate competing risks was performed and the tumor resection was confirmed as an independent risk factor that influences CVD both before PSM and after PSM. Adjustment for cofounding covariates (model 1: age at diagnosis, marital status, PR status and HER2 status) indicated a robust adjusted hazard ratio (HR) of no resection after PSM (in model 1, adjusted HR = 1.165, 95% CI: 1.039–1.306, *P*=0.009). After further adjustment (model 2: all covariates in the baseline), adjusted HR did not change distinctively and the CVD risk among patients without tumor resection increased to 1.166-fold compared to patients with tumor resection (in model 2, adjusted HR = 1.166, 95% CI: 1.040–1.308, *P*=0.009) (**Table 3**, **Supplementary Table S3** and **Table S4**).

**Table 3:** Multivariate competing-risks regression analysis of cardiovascular death.

Variable	Before PSM		After PSM	
	HR (95% CI)	*P* Value	HR (95% CI)	*P* Value
**Unadjusted HR**				
Tumor resection	Reference		Reference	
No resection	1.468 (1.345-1.603)	< 0.001	1.140 (1.017-1.279)	0.025
**Model 1**^a^				
Tumor resection	Reference		Reference	
No resection	1.303 (1.191-1.427)	< 0.001	1.165 (1.039-1.306)	0.009
**Model 2**^b^				
Tumor resection	Reference		Reference	
No resection	1.272 (1.147-1.411)	< 0.001	1.166 (1.040-1.308)	0.009

^a^Model 1: hazard ratios (HRs) were adjusted for statistically significant factors according to univariate analysis (age at diagnosis, marital status, PR status, and HER2 status).

^b^Model 2: hazard ratios were adjusted for all factors in the baseline.

PSM, propensity score matching.

In the univariate competing-risks regression analysis (**Table 2**), the CVD risk between receptor-positive subgroup and receptor-negative subgroup was no statistic difference (all *P*>0.05). In the multivariate analysis (**Supplementary Table S4**), there was also no statistic difference between the positive status and the negative (ER status, HR = 0.980, 95% CI: 0.890–1.079, *P*=0.679; PR status, HR = 0.997, 95% CI: 0.928–1.072, *P*=0.938; HER2 status, HR = 0.905, 95% CI: 0.767–1.069, *P*=0.241).

## Discussion

To the best of our knowledge, this is the first population-based study focusing on CVD risk among breast cancer patients without CT or RT. We found that the CVD risk increased to 2.196-fold in breast cancer patients without CT or RT compared to the general population. Previous SEER-based studies demonstrated that overall breast cancer survivors (including those with or without CT or RT) had higher CVD risk compared to the general population (9, 10). Many previous studies revealed cancer treatment-related cardiotoxicity (28), such as that from CT (6) and RT (7). However, these studies neglected the CVD risk in breast cancer patients who opted out of CT or RT. Interestingly, we found that breast cancer patients who did not receive CT or RT also suffered higher CVD risk than the general population, suggesting that CVD risk in breast cancer patients may increase independently of cardiotoxic therapies. On the other hand, due to lack of detailed information on cardiovascular complications of SEER itself, the other cardiac causes of CVD among patients with breast cancer is unclear besides cardiotoxicity. A recent study on breast cancer related to cardiovascular risk by Greenlee et al. (29) who reported that breast cancer patients suffered from increased risk of cardiovascular related diseases such as cardiac arrest, heart failure, cardiomyopathy, venous thromboembolism and carotid disease, which may be further led to elevated CVD risk.

In accordance with the study by Pavo et al. (30) who found that myocardial damage was directly linked to cancer, and myocardial damage biomarkers were upregulated in cancer patients who did not undergo cardiotoxic therapies. Likewise, a retrospective study revealed that cancer patients without CT or RT had a higher risk of cardiac dysfunction than the age- and gender-matched controls (12). However, these studies were limited due to small sample size, whereas this study included large-scale and multicenter cases. Although cardiovascular comorbidities of the research subjects could not be taken into account in this study, the compared general population also included those with or without cardiovascular comorbidities. The comparison performed by using SMR analysis, which is widely used in similar studies (9, 11, 23) may partly balance out the effect of pre-existing cardiovascular comorbidities. Moreover, the majority of the subjects accepted surgery, which indicated that they had less possibility of having serious cardiovascular comorbidities. These findings highlighted the CVD risk among breast cancer survivors without CT or RT, and the importance of monitoring and preventing CVD in these survivors. We speculate that the tumor itself, not merely the cardiotoxicity of CT or RT, may increase CVD risk among chemoradiotherapy-free patients.

If the speculation that tumors can increase CVD risk is indeed tenable, a reduction of CVD risk may show among breast cancer patients who undergone tumor resection. We found that tumor-resection group had significantly decreased CVD risk compared o tumor-bearing group (tumor resection SMR: 2.031; no resection SMR: 5.425). Nevertheless, the confounding covariates should be taken into account as they may affect the therapeutic approach of choice and the CVD risk, which may cause treatment selection bias and false positive results. To control for this bias, we further utilized PSM to balance the confounding factors and performed competing risk regressions to verify the independent effect of tumor resection on CVD. After PSM, we obtained a robust result, showing that the CVD risk among patients without tumor resection increased by 1.165-fold compared to patients with tumor resection (in model 1, adjusted HR = 1.165; in model 2, adjusted HR = 1.166, both *P*<0.05). The changes in regression coefficients of tumor resection were not obvious after PSM, which indicated that PSM was reliable to balance the basic condition of patients (31). Although we could not completely adjust for all potential confounding variables, we made the greatest efforts to verify our results. These findings should be cautiously interpreted. Our study was consistent with a previous study showing significantly decreased CVD risk for cancer survivors who underwent surgery compared with the no-surgery group (32). However, tumor resection was just a covariate in the previous study (32). The present study provides stronger evidence for the correlation between CVD risk and tumor resection. Our results further support the speculation that existing breast cancer may strongly correlate with higher CVD risk.

Interestingly, even though breast cancer patients were treated with tumor resection, they still had residual CVD risk compared to the general population (SMRs = 1.113–2.037, all *P* < 0.001). This may be attributable to the overlapping risk factors of cardiovascular diseases and breast cancer, such as chronic systemic low-grade inflammation (33), hyperlipidemia, high cholesterol intake, and genetic risk factors (34), which cannot be reversed completely by tumor resection. Further studies on the residual CVD risk among cancer patients are needed.

As far as the impact of receptor status (ER, PR, and HER2) on CVD risk in breast cancer patients is concerned, our results showed that these three receptors had no effect on CVD risk in breast cancer patients. These results were partially consistent with the study by Weberpals, et al. (22) who found that CVD risk was not relevant to the HER2 status and hormone receptor subtypes among breast cancer patients with chemotherapy or radiotherapy. Until now, there is still no direct evidence to show the association between molecular subtypes and CVD outcomes in patients with breast cancer. These results may be affected by the potential cofounding effect of hormonal therapy and targeted therapy involved in these receptors. Further long-term studies of a large sample are needed to focus on the relationship between molecular subtype and CVD risk.

The mechanisms underlying higher CVD risk among breast cancer survivors without CT or RT remain obscure, but the hypothetical explanations may lie in cancer-induced CVD. On the one hand, our PSM-adjusted results support the speculation that breast cancer-bearing patients are at higher CVD risk, whereas the risk could be remarkably reduced after tumor resection. On the other hand, further supporting evidence comes from basic research reporting that tumor-induced inflammation might increase CVD risk. Breast cancer could damage the cardiovascular system of breast cancer-bearing mice by initiating systemic inflammatory reactions named neutrophil extracellular traps (NETs) (35). Cancer-induced NETs could accumulate in the heart and systemic vasculature to induce cardiac dysfunction and vascular dysfunction (13, 36, 37). NETs could also promote cancer-associated arterial thrombosis and further causing ischemic strokes (35, 38). Based on the postulation that tumor-induced inflammation may increase CVD risk, anti-inflammatory drugs should decrease CVD risk. Furthermore, clinical evidences that strengthen our hypothesis come from the Canakinumab Anti-inflammatory Thrombosis Outcomes Study (CANTOS) (39, 40) which showed that anti-inflammatory drugs could reduce both cancer mortality and CVD. In addition, other contributing factors might include cancer-related hypercoagulability (41, 42), tumor metastasis to the cardiovascular system (43), oxidative stress (44) and nitric oxide-dependent endothelial impairment (45). Nevertheless, the interpretations to our data remain speculative and further study is needed to investigate the underlying mechanisms.

### Strengths and Limitations

The strengths of the present study are noteworthy for its large-scale population, long-term follow-up and multicenter cases. To the best of our knowledge, this is the first study to report that breast cancer survivors without CT or RT are inflicted by increased CVD risk on the population level. Some limitations of the study should be taken into account. Firstly, this is a retrospective non-randomized study which may have selection bias for patients. Nevertheless, we used PSM to resolve this limitation as much as possible. Secondly, the data on cardiovascular risk factors, cardiovascular comorbidity, and performance status were not provided by the SEER, and we could not distinguish their effects on CVD risk in our SEER-based study. To address this issue, we used CVD-related SMR in comparing the general population with or without cardiovascular conditions and performance status. Thirdly, the SEER registries do not provide the information of hormonal treatments. Nevertheless, we adjusted the molecular subtype (ER and PR) in multivariate competing-risks analysis, which might partly reduce the effect of hormonal treatments on our results.

## Conclusions

This study found that breast cancer patients free of CT or RT were at higher CVD risk, and tumor resection might be a contributing factor to decreased CVD risk in breast cancer patients. Existing breast cancer might strongly correlate with higher CVD risk. Clinical practices highlight that breast cancer patients free of CT or RT should also be targets for monitoring of CVD risk and prevention of the disease. Clinicians should start to monitor CVD risk and prevent CVD once breast cancer is diagnosed. Further studies and prospective trials are needed to verify our conclusions and to explore the underlying mechanisms.

## Data Availability Statement

The datasets presented in this study can be found in online repositories. The names of the repository/repositories and accession number(s) can be found below: https://seer.cancer.gov/.

## Author Contributions

TG: Conception, study design, data collection, analysis, interpretation of results, figure design, article draft writing, article–review, and editing. HZ and JY: study design, data analysis, interpretation of results, article draft writing, article–review, and editing. WL and KW: interpretation of results and figure design. MS and WP: study design and article draft writing. YML, and YXL: article draft writing. CL: Funding acquisition, interpretation of results, project administration and supervision, article–review, and editing. All authors contributed to the article and approved the submitted version.

## Funding

This study was funded by the National Natural Science Foundation of China (81100235), the Guangzhou Science and Technology Project of China (201804010214), and the Special Funds for the Cultivation of Guangdong College Students’ Scientific and Technological Innovation (“Climbing Program” Special Funds, pdjh2020a0478).

## Conflict of Interest

The authors declare that the research was conducted in the absence of any commercial or financial relationships that could be construed as a potential conflict of interest.

## Abbreviations

AIs, aromatase inhibitors; CIs, confidence intervals; CT, Chemotherapy; CVD, Cardiovascular death; ER, Estrogen receptor; HER2, Human epidermal receptor 2; PR, Progesterone receptor; PSM, Propensity score matching; RT, Radiotherapy; SEER, Surveillance, Epidemiology, and End Result database; SMR, Standardized mortality ratio; SERMs, Selective estrogen receptor modulators.
