# Supplementary material for: Increased Risk of Cardiovascular Death in Breast Cancer Patients Without Chemotherapy or (and) Radiotherapy: A Large Population-Based Study
Source: Front Oncol. 2021 Jan 28;10:619622. doi: 10.3389/fonc.2020.619622 (PMC7876382; doi:10.3389/fonc.2020.619622)
Supplement: Supplementary file 1 [file DataSheet_1.docx]

**Supplementary material**

**Table legends**

**Table S1. Baseline characteristics of 131,306 breast cancer patients.**

^#^Non-White includes Black/American Indian/Alaska Native and Asian/Pacific Islander.

*Low (Grade I: well differentiated and Grade II: moderately differentiated) and high (Grade III: poorly differentiated and Grade IV: undifferentiated).

Abbreviation: ER, estrogen receptor; HER2, human epidermal receptor 2; PR, progesterone receptor.

**Table S2. Age-specific CVD-related SMRs of breast cancer patients in overall, tumor resection and no resection cohort.**

^#^The breast cancer patients without chemotherapy or radiotherapy underwent tumor resection.

*The breast cancer patients without chemotherapy or radiotherapy did not undergo tumor resection.

**Table S3. Regression analysis of multivariate competing-risks for cardiovascular death (Model 1).**

Abbreviation: HER2, human epidermal receptor 2; HR, hazard ratios; PR, progesterone receptor; PSM, propensity score matching.

**Table S4. Regression analysis of multivariate competing-risks for cardiovascular death (Model 2).**

^#^Non-White includes Black/American Indian/ Alaska Native and Asian/Pacific Islander.

*Low (Grade I: well differentiated and Grade II: moderately differentiated) and high (Grade III: poorly differentiated and Grade IV: undifferentiated).

Abbreviation: ER, estrogen receptor; HER2, human epidermal receptor 2; HR, hazard ratios; PR, progesterone receptor; PSM, propensity score matching.**Table S1. Baseline characteristics of 131,306 breast cancer patients**

| **Variable** | **Overall**  **(N/%)** | **No resection**  **(N/%)** | **Tumor resection**  **(N/%)** | ***P* value** |
| --- | --- | --- | --- | --- |
| **N** | 131,306 | 14,294 | 117,012 |  |
| **Age at diagnosis** |  |  |  | < 0.001 |
| 45-60 years | 43,051 (32.8) | 3574 (25.0) | 39,477 (33.7) |  |
| > 60 years | 88,255 (67.2) | 10,720 (75.0) | 77,535 (66.3) |  |
| **Race** |  |  |  | < 0.001 |
| White | 106,054 (80.8) | 10,779 (75.4) | 95,275 (81.4) |  |
| Non-White^#^ | 24,091 (18.3) | 3280 (22.9) | 20,811 (17.8) |  |
| Unknown | 1,161 (0.9) | 235 (1.6) | 926 (0.8) |  |
| **Marital status** |  |  |  | < 0.001 |
| Married | 60,914 (46.4) | 4417 (30.9) | 56,497 (48.3) |  |
| Unmarried | 62,717 (47.8) | 8563 (59.9) | 54,154 (46.3) |  |
| Unknown | 7675 (5.8) | 1314 (9.2) | 6361 (5.4) |  |
| **Laterality** |  |  |  | < 0.001 |
| Left | 66,850 (50.9) | 6,951 (48.6) | 59,899 (51.2) |  |
| Right | 63,642 (48.5) | 6591 (46.1) | 57,051 (48.8) |  |
| Unspecific | 100 (0.1) | 79 (0.6) | 21 (0.0) |  |
| Paired | 714 (0.5) | 673 (4.7) | 41 (0.0) |  |
| **Grade*** |  |  |  | < 0.001 |
| Low | 90,032 (68.6) | 7026 (49.2) | 83,006 (70.9) |  |
| High | 31,255 (23.8) | 3,586 (25.1) | 27,669 (23.6) |  |
| Other/unknown | 10,019 (7.6) | 3682 (25.8) | 6337 (5.4) |  |
| **ER status** |  |  |  | < 0.001 |
| Positive | 106,683 (81.2) | 10,694 (74.8) | 95,989 (82.0) |  |
| Negative | 15,615 (11.9) | 1844 (12.9) | 13,771 (11.8) |  |
| Unknown | 9008 (6.9) | 1756 (12.3) | 7252 (6.2) |  |
| **PR status** |  |  |  | < 0.001 |
| Positive | 91,857 (70.0) | 8863 (62.0) | 82,994 (70.9) |  |
| Negative | 29,031 (22.1) | 3,466 (24.2) | 25,565 (21.8) |  |
| Unknown | 10,418 (7.9) | 1965 (13.7) | 8453 (7.2) |  |
| **HER2 status** |  |  |  | < 0.001 |
| Positive | 6,346 (4.8) | 1,166 (8.2) | 5,180 (4.4) |  |
| Negative | 58,208 (44.3) | 6821 (47.7) | 51,387 (43.9) |  |
| Unknown | 66,752 (50.8) | 6307 (44.1) | 60,445 (51.7) |  |
| **AJCC stage** |  |  |  | < 0.001 |
| I | 73,614 (56.1) | 2,869 (20.1) | 70,745 (60.5) |  |
| II | 40,713 (31.0) | 3,432 (24.0) | 37,281 (31.9) |  |
| III and IV | 16,979 (12.9) | 7993 (55.9) | 8986 (7.7) |  |

**Table S2. Age-specific CVD-related SMRs of breast cancer patients in overall, tumor resection and no resection cohort.**

| **Age** | **Overall** | |  | **Tumor resection^#^** | |  | **No resection*** | |
| --- | --- | --- | --- | --- | --- | --- | --- | --- |
|  | **SMR (95% CI)** | ***P* value** |  | **SMR (95% CI)** | ***P* value** |  | **SMR (95% CI)** | ***P* value** |
| **45-54** | 1.611 (1.343-1.917) | <0.001 |  | 1.489 (1.227-1.790) | <0.001 |  | 4.804 (2.624-8.061) | <0.001 |
| **55-64** | 2.017 (1.830-2.219) | <0.001 |  | 1.811 (1.630-2.007) | <0.001 |  | 6.500 (4.960-8.367) | <0.001 |
| **65-74** | 2.201 (2.079-2.328) | <0.001 |  | 2.037 (1.917-2.163) | <0.001 |  | 5.805 (4.880-6.854) | <0.001 |
| **75-84** | 1.850 (1.786-1.916) | <0.001 |  | 1.767 (1.703-1.833) | <0.001 |  | 3.433 (3.051-3.849) | <0.001 |
| **85^+^** | 1.166 (1.125-1.209) | <0.001 |  | 1.113 (1.070-1.157) | <0.001 |  | 1.603 (1.458-1.758) | <0.001 |
| **Total** | 2.196 (2.148-2.245) | <0.001 |  | 2.031 (1.983-2.079) | <0.001 |  | 5.425 (5.087-5.781) | <0.001 |

**Table S3. Regression analysis of multivariate competing-risks for cardiovascular death (Model 1).**

| **Variable** | **Before PSM** | |  | **After PSM** | |
| --- | --- | --- | --- | --- | --- |
|  | **HR (95% CI)** | ***P* value** |  | **HR (95% CI)** | ***P* value** |
| **Tumor resection** |  |  |  |  |  |
| Yes | Reference |  |  | Reference |  |
| No resection | 1.303 (1.191-1.427) | 0.000 |  | 1.165 (1.039-1.306) | 0.009 |
| **Age at diagnosis** |  |  |  |  |  |
| 45- 60 years | Reference |  |  | Reference |  |
| > 60 years | 11.053 (9.702-12.592) | 0.000 |  | 7.861 (5.825-10.610) | 0.000 |
| **Marital status** |  |  |  |  |  |
| Married | Reference |  |  | Reference |  |
| Unmarried | 2.002 (1.893-2.118) | 0.000 |  | 1.628 (1.413-1.875) | 0.000 |
| **PR status** |  |  |  |  |  |
| Positive | Reference |  |  | Reference |  |
| Negative | 1.012 (0.956-1.071) | 0.690 |  | 0.830 (0.726-0.950) | 0.007 |
| **HER2 status** |  |  |  |  |  |
| Positive | Reference |  |  | Reference |  |
| Negative | 0.891 (0.755-1.051) | 0.170 |  | 0.828 (0.635-1.079) | 0.162 |
| Unknown | 1.320 (1.125-1.548) | 0.001 |  | 1.396 (1.081-1.802) | 0.011 |

**Table S4.** **Regression analysis of multivariate competing-risks for cardiovascular death**

**(Model 2).**

| **Variable** | **Before PSM** | |  | **After PSM** | |
| --- | --- | --- | --- | --- | --- |
|  | **HR (95% CI)** | ***P* Value** |  | **HR (95% CI)** | ***P* Value** |
| **Tumor resection** |  |  |  |  |  |
| Yes | Reference |  |  | Reference |  |
| No resection | 1.272 (1.147-1.411) | 0.000 |  | 1.166 (1.040-1.308) | 0.009 |
| **Age at diagnosis** |  |  |  |  |  |
| 45- 60 years | Reference |  |  | Reference |  |
| > 60 years | 10.914 (9.582-12.431) | 0.000 |  | 7.916 (5.863-10.686) | 0.000 |
| **Race**^#^ |  |  |  |  |  |
| White | Reference |  |  | Reference |  |
| Non-White | 0.893 (0.832-0.958) | 0.002 |  | 0.996 (0.860-1.154) | 0.959 |
| **Marital status** |  |  |  |  |  |
| Married | Reference |  |  | Reference |  |
| Unmarried | 1.975 (1.867-2.090) | 0.000 |  | 1.611 (1.399-1.856) | 0.000 |
| **Laterality** |  |  |  |  |  |
| Left | Reference |  |  | Reference |  |
| Right | 0.964 (0.917-1.013) | 0.148 |  | 0.936 (0.834-1.051) | 0.261 |
| **Grade*** |  |  |  |  |  |
| Low | Reference |  |  | Reference |  |
| High | 1.030 (0.968-1.097) | 0.350 |  | 1.004 (0.875-1.153) | 0.950 |
| **ER status** |  |  |  |  |  |
| Positive | Reference |  |  | Reference |  |
| Negative | 0.980 (0.890-1.079) | 0.679 |  | 1.090 (0.871-1.363) | 0.451 |
| **PR status** |  |  |  |  |  |
| Positive | Reference |  |  | Reference |  |
| Negative | 0.997 (0.928-1.072) | 0.938 |  | 0.814 (0.689-0.963) | 0.016 |
| **HER2 status** |  |  |  |  |  |
| Positive | Reference |  |  | Reference |  |
| Negative | 0.905 (0.767-1.069) | 0.241 |  | 0.839 (0.642-1.098) | 0.201 |
| Unknown | 1.335 (1.138-1.567) | 0.000 |  | 1.445 (1.116-1.871) | 0.005 |
| **AJCC stage** |  |  |  |  |  |
| I | Reference |  |  | Reference |  |
| II | 1.305 (1.236-1.378) | 0.000 |  | 1.182 (1.007-1.387) | 0.040 |
| III and IV | 1.134 (1.033-1.245) | 0.008 |  | 0.874 (0.750-1.019) | 0.086 |
